# The Missing First Step: Underuse of Psychotherapy in Youth Psychiatry

**DOI:** 10.1192/j.eurpsy.2026.10196

**Published:** 2026-07-31

**Authors:** K. Kucharikova, H. Melicharova, J. Vevera

**Affiliations:** 1 Public Mental Health Department, National Institute of Mental Health, Czech Republic, Klecany; 2 Institute of Health Information and Statistics of the Czech Republic, Prague; 3 Department of Psychiatry, Faculty of Medicine in Pilsen, Charles University, Pilsen; 4 Department of Psychiatry, Institute for Postgraduate Medical Education, Prague, Czech Republic

## Abstract

**Introduction:**

Youth mental health is a growing concern, with reports of psychiatric diagnoses becoming increasingly prevalent. In the Czech Republic, the prevalence of adolescent psychiatric diagnoses has increased by approximately 40% over the past decade.

**Objectives:**

While clinical guidelines recommend psychotherapy as the first-line treatment, our nationwide registry study assessed how many pediatric patients received psychotherapy before starting pharmacotherapy.

**Methods:**

We analyzed data from the National Registry of Reimbursed Health Services which covers inpatient and outpatient services as well as prescription medications. We selected patients under 18 years old who were prescribed antipsychotics (ATC group N05A), antidepressants (N06A), anxiolytics (N05B), between 2015 and 2024, separately for every year and every ATC group. We defined the psychopharmaceutical as being prescribed for the first time if the patient had no record of the respective prescription within the previous five years. We then assessed whether patients had received psychotherapy, paid by a health insurance company, within three months prior to the first prescription.

**Results:**

We found an increase in the number of patients under 18 years prescribed antipsychotics and of those prescribed an antidepressant over the study period (14 636 in 2015 vs. 19 648 in 2024 for antipsychotics and 11 145 vs. 21 322 for antidepressants). The number of children and adolescents prescribed these medications for the first time also increased (5 044 in 2015 vs. 5 987 in 2024 for antipsychotics and 5 348 vs. 8 645 for antidepressants). The proportion of patients who attended at least four sessions of psychotherapy, covered by health insurance, within three months prior to the first prescription increased slightly over time from 1.1 % in 2015 to 3.7 % in 2024 for those prescribed antipsychotics, and from 1.6% to 3.9 for those prescribed antidepressants. In contrast, we observed the decrease in the number of children and adolescents prescribed an anxiolytic, decreasing from 36 034 in 2015 to 14 068 in 2024. We observed a similar proportion of decrease in the number of those prescribed an anxiolytic for the first time, from 26 649 patients to 8 384. The proportions of children and adolescents who attended at least 4 sessions of psychotherapy, covered by health insurance, within 3 months prior the first prescription of anxiolytics increased from 0.1 % in 2015 to 1.0 % in 2024.

**Conclusions:**

Our findings indicate insufficient use of psychotherapy. Pharmacotherapy, which for many pediatric diagnoses should be reserved for second-line treatment, was in the vast majority of cases prescribed as the first-line option. These results highlight a critical gap between evidence-based guidelines and real-world clinical practice, underscoring the need for systemic changes to improve access to and utilization of psychotherapeutic interventions in child and adolescent psychiatry.

**Disclosure of Interest:**

None Declared

